# The Uropathogenic Specific Protein Gene *usp* from *Escherichia coli* and *Salmonella bongori* is a Novel Member of the TyrR and H-NS Regulons

**DOI:** 10.3390/microorganisms8030330

**Published:** 2020-02-26

**Authors:** Erik Rihtar, Darja Žgur Bertok, Zdravko Podlesek

**Affiliations:** 1 Biotechnical Faculty, University of Ljubljana, 1000 Ljubljana, Slovenia; erik.rihtar@ki.si (E.R.); zdravko.podlesek@bf.uni-lj.si (Z.P.); 2 National Institute of Chemistry, 1000 Ljubljana, Slovenia

**Keywords:** *usp* promoter expression, TyrR regulon, H-NS nucleoid associated protein, *Escherichia coli*, *Salmonella bongori*, aromatic amino acids, temperature, urea

## Abstract

The *Escherichia coli* PAI*usp* is a small pathogenicity island encoding *usp,* for the uropathogenic specific protein (Usp), a genotoxin and three associated downstream *imu1-3* genes that protect the producer against its own toxin. Bioinformatic analysis revealed the presence of the PAI*usp* also in publically available *Salmonella bongori* and *Salmonella enterica* subps. *salamae* genome sequences. PAI*usp* is in all examined sequences integrated within the *aroP-pdhR* chromosomal intergenic region. The focus of this work was identification of the *usp* promoter and regulatory elements controlling its activity. We show that, in both *E. coli* and *S. bongori*, the divergent TyrR regulated P3 promoter of the *aroP* gene, encoding an aromatic amino acid membrane transporter, drives *usp* transcription while H-NS acts antagonistically repressing expression. Our results show that the horizontally acquired PAI*usp* has integrated into the TyrR regulatory network and that environmental factors such as aromatic amino acids, temperature and urea induce *usp* expression.

## 1. Introduction

*Escherichia coli* and *Salmonella bongori* are both members of the *Enterobacteriacae,* (class gammaproteobacteria) and are presumed to have diverged from a common ancestor 120–150 million years ago [1,2]. The genus *Salmonella* consists of two species, *S. enterica* and *S. bongori,* that have diverged between 40–63.4 Myrs ago [3]. *S. enterica* is divided into six subspecies, *enterica*, *salamae*, *arizonae*, *diarizonae*, *houtenae* and *indica* [1]. While *Salmonella* serovars belonging to *S. enterica,* subspecies *enterica,* provoke disease in humans and other warm-blooded animals, *S. bongori* and the remaining five *S. enterica* subspecies are mostly considered nonpathogenic and commensals of cold-blooded animals. Therefore, only limited research into the remaining five *S. enterica* subspecies and *S. bongori* has been conducted. Nevertheless, *S. bongori* serovar 48:z35 strain RKS3044 is considered endemic in Sicily, Italy and has been reported to infect humans, mainly children aged 1 month to 3 years [4,5]. Furthermore, *S. bongori* strain N19-781 was recently isolated and a draft genome sequence published [6]. Nonetheless, the non-*enterica* subspecies do cause sporadic disease in mammals, with children and immuno-compromised individuals at highest risk [7,8]. On the other hand, *Escherichia coli* is a common commensal inhabitant of the human gastrointestinal tract however, strains that produce virulence factors provoke intestinal as well as extraintestinal infections.

Bacterial virulence factor genes are frequently encoded by pathogenicity islands (PAIs), inserted DNA segments that can be mobile and affect host pathobiology as well as bacterial fitness [9,10]. Gram-negative enteric pathogens have acquired a number of virulence factor genes central for infection via horizontal gene transfer (HGT). During evolution of bacterial pathogenesis, HGT of virulence genes has been a driving force for the emergence of new species and the adaptation of pathogens to new hosts as well as specific niches within their hosts [11].

The *E. coli* uropathogenic specific protein (Usp) is a genotoxin active against mammalian cells and is associated with strains that provoke prostatitis, pyelonephritis and bacteraemia [12,13,14]. Genotoxins have been shown to provoke carcenogenesis by promoting DNA damage in host cells [15,16,17,18]. Studies have found an increased presence of *usp*^+^ *E. coli* strains in colorectal cancer patients [19]. The small pathogenicity island PAI*usp* encodes *usp* and three associated downstream *imu1-3* genes required for protection of the producer against its own toxin.

**Figure 1:**
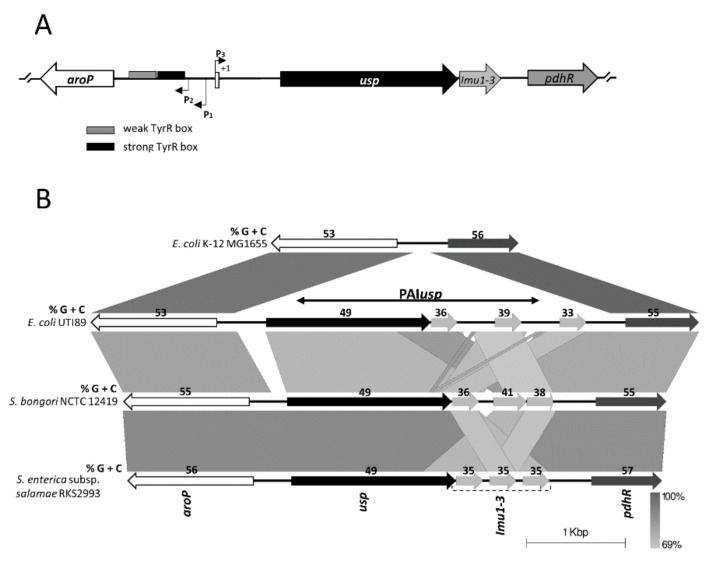
Schematic representation of the *aroP-usp* intergenic region. (**A**), Schematic representation of the *aroP-usp* intergenic region, indicating the position of the TyrR binding boxes and *aroP* promoter sites (not drawn to scale). (**B**) Schematic comparison of the *aroP* and *pdhR* regions in *E. coli* K-12 MG1655, *E. coli* UTI88, *S. bongori* NCTC 12419 and *S. enterica* subsp. *salamae* RKS2993. The graphic was built with Easyfig 2.1 using the blastn algorithm. Similar genes are connected by lines indicative of segments that match in a blastn comparison. Vertical bar at the right bottom indicates the threshold of blastn nucleotide identity values in the alignments. Arrows represent ORFs and are drawn to scale and accurately positioned based on the genome sequences they depict. White, black, light gray and dark gray arrows represent *aroP, usp, imu*1-3 and *pdhR*, respectively. *E. coli* K-12 MG1655 does not harbor PAI*usp*. The G + C content for each of the genes is shown above the arrows.

**Figure 2:**
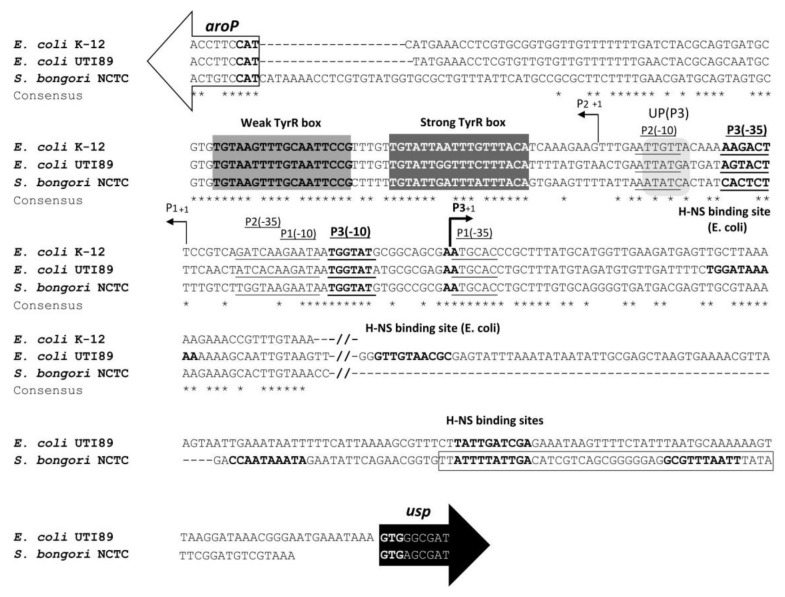
Sequence comparison of the distal *aroP-usp* intergenic regulatory region of *E. coli* K-12 MG1655, *E. coli* UTI89 and S*. bongori* NCTC 12419. Distal regulatory regions from different bacteria were aligned using ClustalW algorithm. Identical nucleotides are indicated below the alignment with an asterisk. Dashed lines represent introduced gaps to maximize the alignment. The distal regulatory region of the *usp* gene, represented by two slashes, differs greatly in UTI89 and *S. bongori*, and thus was not included in this alignment. White and black arrows represent *aroP* and *usp*, respectively. The strong and weak TyrR boxes are shown in grey boxes. Sites similar to the -35 and -10 sites to the *E. coli* K-12 promoters P1 and P2 are underlined, while P3 is underlined and in boldface. UP sequences of promoter P3 (overlapping with P2 -10) are indicated in light grey. The transcription starting point for P1, P2 and P3 is indicated with an arrow and +1. Putative H-NS binding sites, downstream of P3+1, in *E. coli* UTI189 and *S. bongori* NCTC are also in bold. The *S. bongori* region with the putative H-NS binding sites deleted in plasmid pJBTD3 (shown in Figure 3A) is boxed.

Among uropathogenic *E. coli* (UPEC)*,* mostly belonging to the B2 phylogroup, PAI*usp* is located within the *aroP-pdhR* chromosomal intergenic region (Figure 1A). The *aroP* gene encodes an integral membrane protein that transports three aromatic amino acids, phenylalanine, tyrosine and tryptophan into the bacterial cell, while PdhR, the pyruvate dehydrogenase complex regulator controls the *E. coli* respiratory electron transport system. Three *aroP* promoters have been experimentally identified [20,21], P1 the major promoter, P2 located 21 bp downstream of P1, and the divergent P3 which overlaps with P1 (Figure 1A and Figure 2). The P1 promoter is repressed by the transcription regulator TyrR, with phenylalanine, tyrosine or tryptophan, acting as cofactors. The *aroP* gene is thus a member of the TyrR regulon that is involved in aromatic amino acids biosynthesis, catabolism, or transport. Furthermore, TyrR mediated repression of P1 was shown to recruit RNA polymerase to the divergent transcriptionally nonproductive P3 promoter [20,21,22]. The presence of the P3 promoter was proposed to fine tune the required expression levels of *aroP* with a mechanism whereby the substrates of the transporter regulate its synthesis [20,21,22,23].

The primary mechanism of TyrR-mediated gene regulation is repression, nevertheless activation by TyrR was reported for three genes, namely *mtr*, *tyrP* and the *aroP* promoter P3 [23]. A prerequisite for activation activity is a strong, appropriately located TyrR binding box DNA sequence [24], located upstream of the promoter -35 hexamer [23,25]. Members of the TyrR regulon may have two or more TyrR boxes that exhibit different affinities for the TyrR protein and are thus either strong or weak. To the former, TyrR in vitro binds alone, in the absence of aromatic amino acids while binding to the latter occurs only in the presence of tyrosine or phenylalanine and only in the vicinity of a strong box that is on the same face of the DNA helix [23].

H-NS (histone-like nucleoid structuring protein) is a nucleoid associated protein playing a key role in nucleoid organization in Gram negative bacteria. In addition, H-NS is a global regulator of gene expression involved in regulating global transcriptional responses to environmental stress as well as a silencer of xenogenic DNA acquired by horizontal gene transfer [26]. H-NS is a small 15,5 Kd protein that in solution exists as a dimer and binds DNA rich in A T sequences. Following DNA binding, H-NS subsequently oligomerizes and spreads along A T rich DNA mostly repressing gene expression.

In this study, we report the presence of the highly conserved PAI*usp* in *S. bongori* and *S. enterica* subsp. *salamae* strains. In all, PAI*usp* was found to be inserted at the same genomic location as in *E. coli usp*^+^ strains. Furthermore, we investigated and compared *usp* promoter regions and promoter activities from both *S. bongori* and *E. coli*, using reporter gene expression assays as well as Usp protein levels. In both species we identified the *aroP* P3, as the sole promoter of the *usp* gene. Our results also show that the horizontally acquired PAI*usp* has integrated into the TyrR regulatory network and that H-NS represses expression, while environmental factors such as aromatic amino acids, temperature and urea induce *usp* expression.

## 2. Materials and Methods

### 2.1. Bacterial Strains and Plasmids

The strains and plasmids used in this study are listed in Table 1. The *usp* promoter regions were PCR amplified from the uropathogenic *E. coli* strain TA211 which was isolated at the Institute of microbiology and immunology, Medical Faculty, University of Ljubljana, Ljubljana, Slovenia and from the *S. bongori* strain NCTC 12419. The *E. coli* Keio collection strain JW1889 and the *tyrR* defective JW1316 strain, were used throughout the study [27]. Bacteria were routinely grown at 37 °C in LB broth or M9 minimal medium. Media were supplemented with antibiotics when required at final concentrations of 100 µg/mL ampicillin, 50 µg/mL kanamycin or 12.5 µg/mL tetracycline (all from Sigma-Aldrich, St. Louis, MO, USA). To study promoter regulation, the L-aromatic amino acid phenylalanine was added to the M9 minimal medium at a final concentration of 1 mM. Freshly prepared urea stock was added to the M9 medium at a final concentration of 0.2, 0.4 or 0.6 M. To impose salt stress, 0.3 M NaCl was added to M9 minimal medium.

### 2.2. Computational Analysis and Genomic Island Sequence Comparisons

Genome mining for Usp homologs was carried out by PSI-BLAST and tBLASTn analysis using the *E. coli* UTI89 Usp protein (accession no. ABE05631.1) as queried against the NCBI non-redundant protein database. For phylogenetic analysis, multiple sequence alignments (restricted to complete genomes from NCBI Genome Information resource, July 2018) were based on a ClustalW program implemented in MEGA7 software [29], using the default settings. A neighbor-joining phylogenetic tree of Usp homologs from different bacterial species was generated using MEGA7.

The genomic regions of interest for comparison analysis were manually extracted from genomes of *E. coli* K-12 MG1655 (RefSeq NC_000913), *E. coli* UTI89 (RefSeq NC_007946.1), *S. bongori* NCTC 12419 (RefSeq NC_015761.1) and *S. enterica* subsp. *salamae* RKS2993 (RefSeq NZ_JXTT01000055). To compare genomic regions of the four strains, we performed nucleotide alignment using EasyFig version 2.1 [30], with the blastn algorithm with minimum length of hits set to 50 and maximum E- value set to 0. 001. 

A basic comparative analysis of protein or nucleotide sequences was carried out using ClustalW version 2.0 [31]. Conserved protein domains encoded by the PAI*usp* were determined by cross reference of the Conserved Domain Database and Search Service version 3.16 [32].

### 2.3. Recombinant DNA Manipulations

All cloning was performed as described previously [33]. Plasmid DNA was isolated using the GeneJET plasmid miniprep kit (Thermo Fisher Scientific). Restriction enzymes and T4 DNA ligase (all Thermo Fisher Scientific) were used as directed by the manufacturers. PCR amplifications were carried out using Vent DNA polymerase (New England Biolabs). PCR and restriction fragments were purified using PCR purification kit (Thermo Fisher Scientific).

For *usp*-CAT reporter transcription fusions, the promoter region was PCR amplified from the *S. bongori* NCTC 12419 chromosomal DNA using the Bong_L_Pst_F and Bong_L_Bst_R primer pair (Table 2). The PCR product was cloned into the pJET1.2 cloning (high copy) plasmid (Thermo Fisher Scientific). The newly created *Pst*I restriction site was then utilized to remove the upstream T7 promoter present in the vector. Digestion with *Pvu*II and subsequent religation removed the downstream P_lacUV5_ promoter. This intermediate plasmid was designated pJBPLN3. The promoterless chloramphenicol resistance gene was amplified from the pLysS vector using primers Cm_Bst_F and Cm_Xba_R, with flanking *Bst*EII and *Xba*I restriction sites. The digested PCR product was ligated into pJBPLN3, also digested with the same two enyzmes. The resulting plasmid, pJBLCm, was subsequently used in the construction of all *usp* promoter fragments from either *S. bongori* or *E. coli*, carrying different promoter variants by replacing the *Pst*I and *Bst*EII promoter fragment.

For *usp-lacZ* reporter fusions, PCR amplification was performed using primers listed in Table 2. Promoter fragments were then cloned into the low copy *lacZ* expression vector pRW50 [28]. Site-directed mutagenesis of the -10 or -35 promoter sequences was performed with synthetic primers as described by [34]. Mutations were confirmed by DNA sequence analysis.

### 2.4. Minimal Inhibitory Concentrations (MICs) for Chloramphenicol

All of the plasmids harboring promoter fragments (Table 1) were transformed into the *E. coli* K-12 MG1655 strain. The chloramphenicol MIC were determined using the broth microdilution method. Briefly, overnight cultures were diluted in fresh LB medium (1:4000) and subsequently 0.1 mL of the diluted culture was inoculated into a series of tubes with increasing concentrations of chloramphenicol and grown for 24 h. Background MIC values from cells carrying a promoterless pJCAT, were subtracted.

### 2.5. β-Galactosidase Assays

Plasmids harboring promoter fragments (Table 1) were transformed into the relevant bacterial strains. The overnight cultures were diluted in either fresh M9 minimal or LB medium (1:100) and grown at 37 °C for 24 h. Subsequently, cells were collected (1 mL) by centrifugation. β-galactosidase activity was assayed according to Miller, 1974 [35]. The presented data are the results of at least three independent experiments and are shown with standard deviations. Background β-galactosidase activity values, generated form cells carrying a promoterless pRW50, were subtracted.

### 2.6. Western Immunoblotting

*E. coli* K12 MG1655 strain, transformed with plasmids pUSP4L (containing UPEC *usp* gene under native P3 promoter) and pCUPBI (containing UPEC *usp* gene under native *S. bongori* P3 promoter), were grown at the designated temperatures with shaking in LB medium. After 24 h the cells, normalized to optical density, were collected by centrifugation. N-terminally His_6_-tagged Usp was affinity purified by Ni-NTA metal affinity chromatography (Qiagen, Germany) and eluted with 200 µl of buffer C (50 mM NaH_2_PO_4_ pH 8, 300 mM NaCl, 250 mM imidazole, 20 mM mercaptoethanol). Proteins in eluate were precipitated with TCA and washed with ice-cold acetone. The pellet was dried and resuspended in SDS loading buffer, separated on 12% SDS-PAGE gel and transferred at 200 mA for 90 min onto a PVDF membrane (Millipore, U.S.A.) for immunoblotting. The membrane was blocked in Tris-Buffered saline with 0.1% Tween 20 (TBS-T) containing 5% (w/v) bovine serum albumin (BSA). The membrane was subsequently incubated for 2 h with mouse anti-penta-histidine antibodies (Thermo Fisher Scientific) for anti-His staining, followed by 1 h incubation with horseradish peroxidase-conjugated rabbit anti-mouse IgG. The protein bands were visualized by an enhanced chemiluminescence kit (ECL; Amersham).

### 2.7. Protein Isolation and Electrophoretic Mobility Shift Assay (EMSA)

To isolate the purified TyrR and H-NS proteins, *E. coli* BL21 (DE3) cells containing the pET8HT or pET9HT expression plasmids were grown in LB medium and induced with 0.8 mM of isopropyl β-D-thiogalactopyranoside (IPTG) for 4 h at 37 °C. N-terminally His_6_-tagged TyrR protein was affinity purified by Ni-NTA metal affinity chromatography (Qiagen). The native purification protocol was followed according to the manufacturer’s instructions. Protein purity was assessed on a 10% SDS-PAGE gel, followed by Coomassie blue staining and the eluate was dialyzed overnight against a buffer (50 mM Tris-HCl pH 7.4, 150 mM NaCl, 5 mM dithiothreitol DTT, 10% glycerol) at 4 °C. The concentration of the purified TyrR protein was determined using NanoDrop 1000 (Thermo Fisher Scientific) with the extinction coefficients at 280 nm of 36,245 M^−1^ cm^−1^.

The reactions for EMSA assay were carried out as described previously with modifications [22]. Binding reactions were performed by mixing DNA fragments (~15 nM) with the purified His_6_-tagged TyrR protein (~15 nM), in a total volume of 10 µl of binding buffer (50 mM Tris-HCl pH 7.8, 50 mM NaCl, 3 mM magnesium acetate, 0.1 mM EDTA, 0.1 mM dithiothreitol (DTT), 25 µg/mL bovine serum albumin (BSA)) with added phenylalanine (0.1 mM) and ATP (0.1 mM). Protein-DNA binding reactions were incubated at 37 °C for 30 min and then analyzed on 2% agarose gel (2% agarose, 1% glycerol, 0.5x Tris - borate –EDTA, 1 mM MgCl_2_, 0.1 mM ATP, 0.1 mM phenylalanine). Protein-DNA complexes were resolved at 60 volts at 4 °C. The DNA fragments were stained with ethidium bromide and visualized. Binding reactions with the purified H-NS protein were performed as described previously [36].

Usp protein levels were quantified using GeneTools (Syngene International Ltd.).

### 2.8. Statistical Analysis

Data analyses were performed using GraphPad PRISM version 6.01 software. Statistically significant differences in gene expression from each promoter were determined by two-way analysis of variance (ANOVA) with post-hoc comparisons using the Tukey multiple comparison test.

## 3. Results

### 3.1. Bioinformatic Analysis of PAIusp prevalence

Whilst the PAIs of *E. coli* generally have a size of 30–100 kb and are associated with genes for tRNA, encode integrases and are flanked by direct repeats, *usp* is encoded on a small approximately 4 kb pathogenicity island, encoding only *usp* and the tightly associated *imu* genes. Our analysis showed that PAI*usp* is in all publicly available *usp^+^ E. coli* genome sequences, inserted into the intergenic region *aroP-pdhR*. The *aroP* gene encodes the aromatic amino acid permease and *pdhR,* the pyruvate dehydrogenase complex regulator. Interestingly, bioinformatic analysis revealed the presence of PAI*usp* homologs in all publicly available *Salmonella bongori* genomes, including the clinical isolate N268-08 [37] and in *Salmonella enterica* subsp. *salamae* genomes, inserted at exactly the same genome position as in all *usp*^+^*E. coli* genomes (Figure 1 and Table S1).

It has been reported that the Usp protein from *E. coli* strains harbors an N-terminal Hcp domain (PF05638), an extended C-terminal S-type pyocin domain (PF06958) and a colicin nuclease domain (PF12639) [38]. Pairwise alignment of the Usp proteins of *E. coli* and *S. bongori* strains, revealed ~76% sequence identity. Furthermore, the additional ORFs encoding Usp homologs in *Salmonella enterica* subsp. *salamae* strains show protein sequence identity of ~82% and ~77% compared to Usp from *S. bongori* strains and *E. coli* strains, respectively. All of the above-mentioned proteins from the various bacteria have the same domain structure as the Usp from *E. coli* (Figure S1A and Figure S1B). Phylogenetic analysis revealed that the Usp protein derived from *E. coli* clearly segregates as a separate lineage in the NJ tree from *Salmonella* which in turn splits into two clades, *S. bongori* and *S. enterica* subsp. *salamae* (Figure S1C).

Further analysis revealed that the *E. coli usp* flanking genes, *aroP* and *pdhR,* have a total G + C content (55%) similar to the average G + C content of an *Escherichia* genome (~51%) (indicating, that these are ancestral genes). On the other hand, all *usp* and associated immunity protein genes (*imu*1-3) have considerably lower G + C contents (49%, 36%, 38%, 38%, respectively) indicating acquisition via horizontal gene transfer (Figure 1B). G + C analysis showed similar results for Usp homologs in *S. bongori* and *S. enterica* subsp. *salamae*.

Herein we investigated activities of the *E. coli* and *S. bongori usp* promoters. Since data on *usp* expression were lacking, we initially set to in silico identify the *usp* promoter region. Sequence alignment of the *aroP-usp* intergenic regions of *E. coli* UTI89 and *S. bongori* NTCT 12419, revealed significant differences in the upstream proximal region (>200 bp relative to the *usp* start codon) (Figure 2). As in both genomes, we were unable to identify -35 (TTGACA) and -10 (TATAAT) consensus sequences [39], we speculated that the previously identified divergent *aroP* promoter P3 [20,21], located in the *usp* upstream distal region, could drive PAI*usp* transcription. In the aligned sequences of *E. coli* UTI89 and *S. bongori* NTCT 12419, the P3 promoter is located at exactly the same position as in *E. coli* K-12. The P3 -10 site is conserved in all available sequences, while the -35 sites from *S. bongori* and UTI89 differ compared to the commensal *E. coli* strain K-12 (without PAI*usp*), in three and two bases, respectively (Figure 2).

### 3.2. Expression of usp from the Divergent aroP P3 Promoter

**Figure 3:**
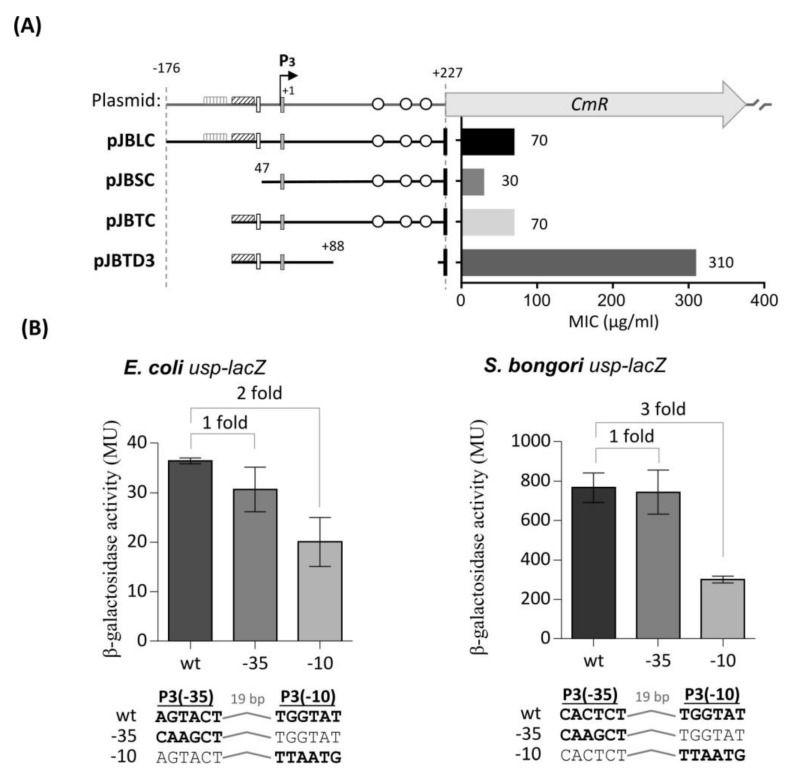
P3 promoter activity from both *E. coli* UPEC and *S. bongori*. (**A**) Analysis of P3 promoter activity from *S. bongori* using promoter probe plasmid pJCAT. The tested DNA fragments and their locations relative to the transcription start site +1 of P3 are shown. The numbers refer to nucleotides. Boxes indicate strong (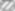) and weak (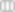) TyrR boxes. The UP element is designated as an open square and H-NS binding sites as open circles. The ability of the fragments to drive CAT expression in *usp*-CAT transcriptional fusions was determined indirectly by measuring MIC of chloramphenicol. (**B**) Effects of site-specific mutations in either the -35 or -10 site of P3. Units for β-galactosidase assay are those defined by Miller, 1974 [35]. The values of β-galactosidase activities are averages of three independent experiments, with standard deviations below 15%. Values above columns are fold repression: specific activity of β-galactosidase of the wild-type promoters divided by that of site-specific mutated promoters.

Initially, promoter activity of DNA fragments from the *S. bongori* NCTC 12419 *aroP-usp* intergenic region, was investigated using the promoter-probe pJBLC plasmid with a chloramphenicol acetyl transferase reporter gene (CAT). Fragments with promoter activity drive CAT expression, resulting in resistance to chloramphenicol (Figure 3A). CAT assays were performed indirectly by measuring MIC of chloramphenicol. The DNA fragment encompassing the entire intergenic region (pJBLC, 403 bp) supported expression (MIC 70 µg/mL) in *E. coli* strain MG1655. On the other hand, MIC of chloramphenicol from a shorter DNA fragment (pJBSC, 274 bp), without the two (weak and strong) TyrR binding boxes, was approximately two fold lower, compared to that of the full length fragment indicating, that the TyrR protein acts as a transcription activator of the *S. bongori* P3 promoter. Furthermore, no significant effect on MIC of chloramphenicol was observed, if only the weak TyrR box was excluded (pJBTC, 315 bp). The smallest DNA fragment that could support significant expression was the 111 bp region harboring the strong TyrR box and the intact P3 promoter (not shown). Interestingly, when the 111 bp DNA fragment was extended for 64 bp (pJBPTD3), MIC of chloramphenicol was, in comparison to the full-length fragment (pJBLC), 4.4 fold higher. The same observation was made by Wang et al. (1997b) for the *E. coli aroP* promoter P3. They proposed that *E. coli* P3 and P1 have AT rich associated elements which could function as UP sequences and confer higher transcription levels (Figure 2). Bacterial UP elements are A T rich regions upstream of the -35 promoter element that enhance/activate transcription through contact with the alpha subunit carboxy-terminal domain of the RNA polymerase core enzyme [40,41].

Subsequently, we studied and compared *aroP* P3 promoter activity from *E. coli* and *S. bongori* strains on the basis of β-galactosidase activity from *usp-lacZ* transcription fusions (Figure 3B). Under the tested conditions we observed ~20 fold higher β-galactosidase activity from the *S. bongori* P3 compared to that from *E. coli*.

In an attempt to clarify the basis of the pronounced difference between *S. bongori* and *E. coli* P3 promoter activity, the effect of modifications in the -35 and -10 sites were investigated from the low copy number plasmid pRW50 (Table 1). The -10 site (wild type TGGTAT) was in both *S. bongori* and *E. coli* promoters altered to TTAATG, while the -35 site was in both promoters altered (wt. in *S. bongori* and *E. coli*, CACTCT and AGTACT, respectively) to CAAGCT (Figure 3B). The modifications introduced into the -10 region reduced P3 activity of both *S. bongori* and *E. coli* promoters, 3 fold (*p*-value < 0.0001) and 2 fold (*p*-value 0.008), respectively. On the other hand, modifications of the -35 regions exhibited no significant effect on promoter activity (Figure 3B). Thus, we conclude that the -10 site of promoter P3 plays a significant role in *usp* expression and furthermore, the observed difference in the level of transcription activity between the *S. bongori* and *E. coli* is not due to differences in the P3 -35 sites.

### 3.3. Positive Regulation of the usp P3 Promoter

In *E. coli* K-12, which does not harbor PAI*usp*, the P3 promoter was previously shown to exhibit very low-level activity [20]. To ascertain whether the *aroP* P3 promoter from *E. coli* and *S. bongori,* can be activated by aromatic amino acids via TyrR binding to the TyrR boxes, expression from the P3 *usp*-*lacZ* fusion was investigated in the *tyrR*^+^ (JW1889) and in the t*yrR^-^* defective strain (JW1316) in minimal medium with or without phenylalanine.

**Figure 4:**
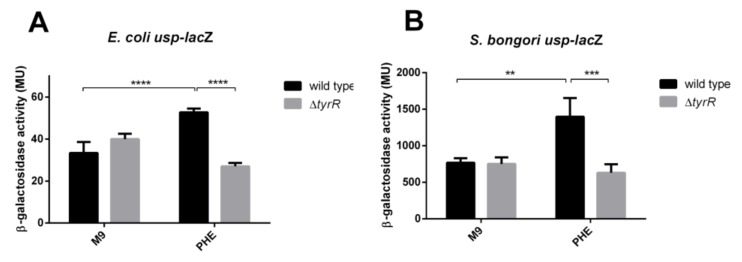
Expression of *usp* from P3 is modulated by the TyrR protein. Transcription from *S. bongori* (**A**) and *E. coli* (**B**) P3 in the presence or absence of aromatic amino acid phenylalanine in wild-type *tyrR*^+^ (black bars) and *tyrR*^−^ null phenotype strain (grey bars). Error bars represent standard deviation of the means of three independent experiments. M9, minimal medium; PHE, M9 supplemented with 1mM phenylalanine. Significant differences between or among groups are indicated, ** for *p* < 0.01, *** for *p* < 0.001 and **** for *p* < 0.0001 respectively.

Our results showed that in the *tyrR^+^* strain, the aromatic amino acid phenylalanine provoked a statistically significant increase in expression from both the *S. bongori* and *E. coli* P3 promoters 2-fold (*p*-value 0.0021) and 1.6 fold (*p*-value < 0.0001), respectively (Figure 4). On the other hand, in the *tyrR-* defective strain, no aromatic amino acid mediated activation of P3 promoter activity was detected (*p*-value 0.6641 in *S. bongori* and *p*-value 0.1097 in *E. coli*) indicating, that binding of the TyrR protein to the P3 upstream region, is critical for activation and regulation.

### 3.4. TyrR Binding to the S. bongori aroP-usp TyrR Boxes.

**Figure 5:**
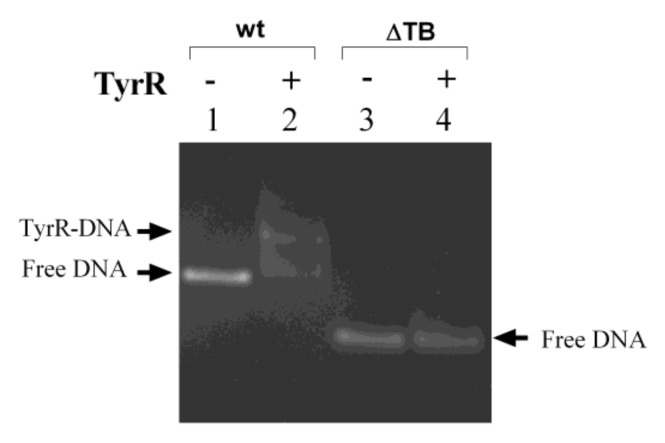
Electrophoretic mobility assay (EMSA) showing binding of the TyrR protein to the TyrR boxes within the *S. bongori aroP-usp* intergenic region. The DNA fragments corresponding to the wild type (wt, 403 bp) *aroP-usp* intergenic sequence (Lanes 1-2) and shorter DNA fragment without TyrR binding boxes (ΔTB, 274 bp) (Lanes 3-4). The DNA fragments, wt and ΔTB were each incubated in the presence or absence of TyrR protein (14 nM). The positions of free DNA and TyrR-DNA complexes are marked.

Previously, Wang et al., 1997a,b [20,21] demonstrated TyrR protein binding to the TyrR boxes within the intergenic region of *aroP-pdhR* in *E. coli* strain K-12. Here we demonstrated that the TyrR protein also specifically binds to the TyrR boxes within the *S. bongori aroP-usp* intergenic region. Incubation of the purified His_6_-tagged TyrR protein, with a DNA fragment encompassing the wild type *aroP-usp* intergenic region (403 bp) provoked, a mobility shift consistent with TyrR-DNA complex formation (Figure 5, Lanes 1 and 2). To prove unequivocally that binding of TyrR to the DNA fragment is specific, a control experiment was performed using the DNA fragment without TyrR binding boxes (274 bp) and no TyrR-DNA complex was observed (Figure 5, Lanes 3 and 4).

### 3.5. Repression of E. coli and S. bongori usp Expression by H-NS

To ascertain whether other transcription factors are involved in regulation of *usp* activity, an in silico search was performed using Virtual footprint [42]. Three putative H-NS binding sequences were found in the *E. coli* and *S. bongori* promoter regions. To corroborate the involvement of H-NS in regulation of *usp* expression, we subsequently investigated activity of P3 *usp-lacZ* fusions from *E. coli* and *S. bongori* as well as Usp protein levels in an *hns*^+^ (JW1889) and *hns^-^* defective *E. coli* strain (JW1225).

**Figure 6:**
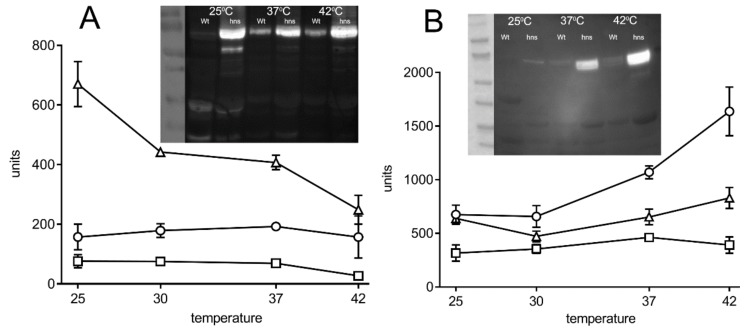
Expression of *E. coli* and *S. bongori usp* genes in wild type (○), *tyrR* (□) and *hns* (Δ) defective strains at different temperatures. (**A**) Expression driven by the *E. coli usp* promoter. Expression from the *usp-lacZ* fusion of β-galactosidase and the inset shows the Western blot analysis of Usp protein levels in wild type and *hns* defective strains; (**B**) Expression driven by the *S. bongori usp* promoter, expression from *usp-lacZ* and inset, Usp protein levels; ○ wild type strain; □ *tyrR* mutant strain and Δ *hns* defective strain. Error bars represent standard deviations of the means of three independent experiments. Usp protein levels were determined at least three times and representative results are shown.

Our results showed higher activity of both *usp-lacZ* fusions as well as higher Usp protein levels in the *hns^-^* defective *E. coli* strain compared to the wild type indicating that H-NS acts as a repressor of *usp* expression (Figure 6). As temperature is an important signal regulating bacterial gene expression and as *S. bongori* is mostly considered restricted to cold-blooded animals we investigated *usp* promoter activity and Usp protein levels following growth at 25 °C, 30 °C, 37 °C, and 42 °C, in the wild type and in the *hns* mutant strain. Temperature dependent synthesis of the Usp protein was observed from the *E. coli* and *S. bongori* promoters. While under the tested conditions, *usp-lacZ* expression revealed higher *S. bongori usp* promoter activity compared to the *E. coli* promoter, protein levels from the latter were at all investigated temperatures in the w.t. and *hns* mutant strain significantly higher (Table S2).

From the *E. coli* promoter, the wild type strain produced approximately 5 fold higher levels of Usp at 37 °C and 42 °C than at 25 °C while in the *hns* mutant, Usp levels were higher and comparable at all three temperatures. From the *S. bongori* promoter, Usp levels were in the wild type strain grown at 37 °C and at 42 °C approximately eight fold higher than when grown at 25 °C while in the *hns* mutant strain, protein levels were at 37 °C and 42 °C higher, approximately 9 and 16 fold, respectively, than when cultivated at 25 °C. Our results indicate that in *E. coli* H-NS plays a key role in temperature dependent *usp* expression. Nevertheless, discrepancies between protein levels and promoter activities from the *E. coli* and *S. bongori usp-lacZ* fusions are evident. From the *E. coli usp-lacZ* fusion temperature dependent expression was observed in the *hns* defective strain indicating that other factors are also involved.

### 3.6. H-NS binding to usp Promoter Region

**Figure 7:**
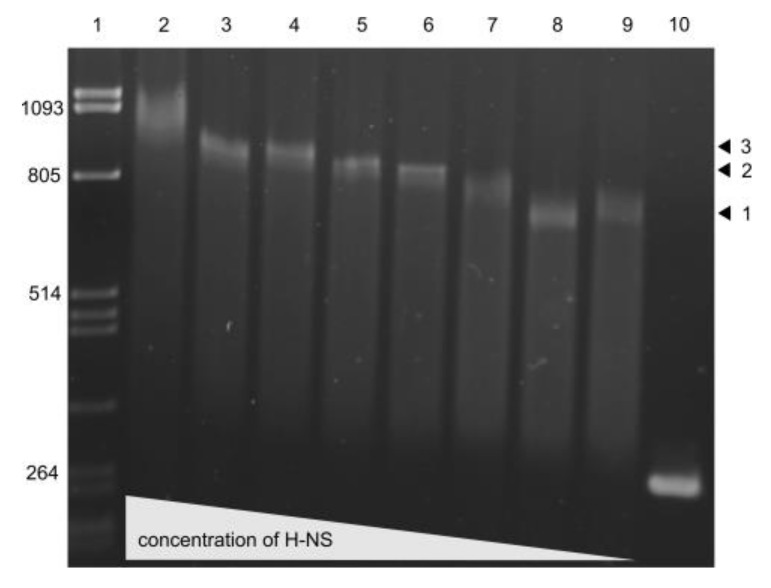
Electrophoretic mobility assay (EMSA) showing binding of the H-NS protein to the H-NS binding sites within the *S. bongori usp* promoter region. Lane 1, *Pst*I digested λ DNA; Lanes 1-9 *usp* promoter fragment with H-NS binding sites and increasing concentrations of purified H-NS protein (17.5 nM–1.6 nM); Lane 10, free DNA. Arrows 1, 2, and 3 designate protein concentration dependent increase in DNA shifts due to H-NS binding to 1, 2 or 3 binding sites.

Our in silico analysis revealed three putative H-NS binding sites in the *usp* promoter regions (Figure 2). To assess H-NS binding, EMSA experiments were performed following incubation of the promoter fragment harboring the three binding sites with various concentrations of the purified H-NS protein (from 17.5 nM to 1.6 nM). As shown in Figure 7, increasing protein concentrations provoked a step wise increase in DNA shifts with a full shift observed at 17.5 nM H-NS due to binding to all three sites.

### 3.7. Urea Mediated Activation of Promoter P3

As PAI*usp* is prevalent among uropathogenic *E. coli*, that likely encounter osmotic stress in the urinary tract during infection, we postulated that urea and possibly another osmolyte NaCl, could affect *usp* expression from the *aroP* P3 promoter in *E. coli* and *S. bongori*. Expression from both, UPEC and *S. bongori*, *usp-lacZ* fusions was therefore investigated under urea induced stress in the *tyrR*^+^ and the *tyrR^-^* defective strain. All cultures were grown for 24 h in M9 minimal medium and in M9 minimal medium supplemented with 0.4M, 0.6M urea or 0.3 M NaCl, concentrations that are comparable to those in human urine [43]. To ascertain whether the TyrR transcription regulator is also involved in urea mediated regulation of *aroP* P3, β-galactosidase activity was also investigated in the *tyrR^-^*defective strain.

**Figure 8:**
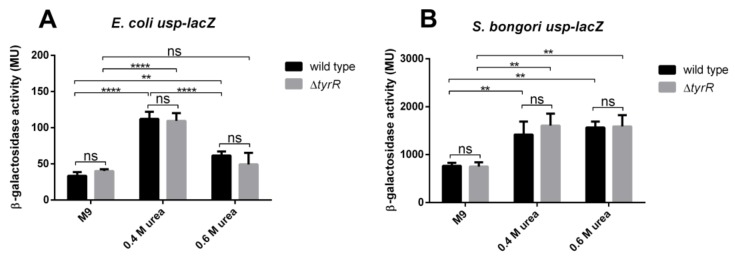
Expression of *usp* from P3 promoter is mediated by urea. Transcription from (**A**) *S. bongori* and (**B**) *E. coli* P3 in the presence or absence of urea in wild type *tyrR*^+^ (black bars) and in *tyrR*^−^ defective strain (grey bars). Units for β-galactosidase assay are those defined by Miller, 1974. Error bars represent standard deviations of the means of three independent experiments. M9, minimal medium; M9 supplemented with 0.4 M urea; M9 supplemented with 0.6 M urea. Significant differences between or among groups are indicated, ns for > 0.05, ** for *p* < 0.01 and **** for *p* < 0.0001.

Our results showed statistically significant increases in β-galactosidase activity from *E. coli* and *S. bongori* promoter fusions in the presence of 0.4 M urea compared to a medium without urea, 3.5 (*p*-value < 0.0001) and 2.1 fold (*p*-value 0.0092), respectively (Figure 8). However, the effect of urea was independent of TyrR mediated regulation of promoter P3 (*p*-value > 0.5). Induction of *S. bongori usp* promoter activity was comparable in the presence of 0.6 M urea and 0.4 M urea. Surprisingly, we observed an ~1.8– fold (*p*-value 0.0001) reduction of *E. coli* P3 activity in the presence of 0.6 M urea compared to 0.4 M urea. On the other hand, 0.3 M NaCl provoked no significant increase in P3 promoter activity (data not shown).

## 4. Discussion

Here we present a study of the dissemination and regulation of the horizontally acquired PAI*usp*, *usp* promoter activity. Point mutations and DNA rearrangements are significant for evolution and speciation of bacterial species nevertheless HGT, the ability to acquire foreign genetic material, enables much more rapid adaptation and survival in novel ecological niches [9,44]. HGT occurs in all free-living bacteria and has played a major role in acquisition of virulence factor and antibiotic resistance genes [45,46]. A paradigm of HGT in bacterial speciation is the divergence of *E. coli* and *Salmonella* spp., that presumably occurred approximately 140 million years ago [2].

Our analysis of publicly available genome sequences, indicated limited dissemination of the PAI*usp.* Interestingly, *S. bongori* was previously shown to have effectors with virulence associated homologues in enteropathogenic *E. coli* and enterohemorrhagic *E. coli* [47]. The presence and identical genomic location in *E. coli*, *S. bongori* and *S. enterica* subsp. *salamae* strains indicates a common yet unknown ancient source and that acquisition via HGT occurred only once, or alternatively, that the *aroP-pdhR* intergenic region represents a specific integration site. Regulatory systems control gene expression and genes acquired via HGT must be in the new host appropriately expressed. While exogenous sequences can be eventually incorporated into preexisting regulatory networks, expression may initially be downregulated by specific “xenogenic silencing proteins”, such as the H-NS family of proteins and others, that bind to AT-rich DNA [41]. Further, while expression of horizontally acquired genes is in a number of pathogens essential during infection, outside the host expression could be detrimental. Additionally, mutations in regulatory regions affect expression of virulence factor genes and thus pathogen evolution. Similarly, in *E. coli* and *S. bongori* the horizontally acquired PAI*usp* gene cluster appears to have evolved new regulation after transfer and integration into the *aroP-pdhR* intergenic region, enabling expression of an otherwise silent *aroP* promoter P3. Thus, while *usp-lacZ* fusions showed significantly higher levels of expression from the *S. bongori* P3 promoter compared to that of *E. coli* P3, higher Usp protein levels from the latter could indicate more efficient translation possibly, to fine tune *usp* expression in particular species and subspecies inhabiting distinct ecological niches. Furthermore, a number of differences in the *E. coli* and *S. bongori* regulatory regions, such as position of and subtle differences in H-NS binding sequences as well as possibly in DNA topology, could be responsible for the observed higher expression of the *S. bongori usp-lacZ fusion*.

Integration of virulence factor genes acquired by HGT often does not suffice to promote pathogenicity or to exhibit a newly acquired phenotype [48,49,50] possibly due to the lack of appropriate gene regulation or even the absence of a promoter region. Thus, evolution of regulation of acquired genes has broader implications for our understanding of HGT and evolution of virulence. Here we demonstrated that PAI*usp* usurped the divergent TyrR regulated *aroP* P3 promoter resulting in adjustment of PAI*usp* gene expression with amino acid availability in the environment. This process required specific promoter mutations which enabled activation of the otherwise silent promoter P3.

In our study TyrR in conjunction with aromatic amino acids, induced a significant increase in *usp* expression in both bacteria, making *usp* a new member of the TyrR regulon. This is the first report of TyrR mediated expression of a virulence gene in *E. coli* and *S. bongori*, extending the significance of the TyrR regulon. Nonetheless, evidence indicates that TyrR [23] is directly or indirectly involved in virulence of other pathogens namely, it has been shown to be required for *Yersinia pestis* pathogenesis and extracellular survival/proliferation [51]. Additionally, two aromatic pathway genes (*aroE* and *aroA*) play a crucial role in *Y. pestis* fitness in deep tissues during infection [52]. Aromatic amino acid biosynthesis has also been shown to be required for the intracellular replication of *Listeria monocytogenes* and *Shigella flexneri* [53,54].

H-NS is a repressor of a number of virulence factor genes frequently encoded on horizontally acquired pathogenicity islands, preventing their expression under non-permissive conditions, i.e., outside the host. The protein consists of an N terminal dimerization domain, a second, central dimerization domain and a C-terminal domain for DNA binding. The N terminal domain harbors four α-helices that allow self-association by contacts “head-to-head” and “tail-to-tail”. These interactions may occur simultaneously allowing H-NS oligomerization to form nucleoprotein filaments that are either bridged or linear. H-NS compacts DNA, repressing expression particularly at the level of transcription [55,56,57]. Our results demonstrated that H-NS is a repressor of Usp synthesis as protein levels were higher from the *E. coli* and *S. bongori* native promoters in a *hns* defective strain compared to the wild type. In accordance, *usp-lacZ* expression from the *E. coli* promoter was also higher in the *hns* mutant but, in contrast, expression from the *S. bongori* promoter was lower. These results indicate that in *S. bongori* additional, possibly indirect posttranscriptional mechanisms involving H-NS could regulate Usp synthesis. Furthermore, deletion of the promoter region harboring the putative H-NS binding site, significantly increased *usp* promoter activity as demonstrated by an increase in MIC for chloramphenicol from plasmid pJBPTD3 (Figure 3A). Temperature is an important signal regulating bacterial gene expression and H-NS has been previously shown to be a key regulator in responses to environmental stimuli including temperature, enabling bacteria to differentiate within host vs. ambient environment [58,59]. Therefore, *usp* promoter activity and Usp protein levels were investigated and compared following growth at a number of temperatures. Indeed, while from the *E. coli* promoter the wild type strain produced approximately 5 fold higher levels of Usp at 37 °C and 42 °C than at 25 °C, in the *hns* mutant, Usp levels were comparable at all three temperatures indicating, that in *E. coli* H-NS plays a significant role in temperature dependent *usp* regulation. At the molecular level, temperature provokes alterations in local DNA structure [60] as well as alterations in the H-NS protein [61]. Nevertheless, discrepancies are evident, as from the *E. coli usp-lacZ* fusion temperature dependent expression was observed in the *hns* defective strain indicating that, at the level of promoter activity, other as of yet unresolved factors, are also involved. Additional studies will be performed to further unravel the complex molecular mechanisms regulating *usp* expression in *E. coli* and *S. bongori* in response to environmental cues.

Urea freely penetrates cell membranes and destabilizes protein conformation [62], however no *E. coli* receptor or transcription regulator that responds to urea has been identified. As UPEC colonize the urinary tract they are exposed to high osmolality, due to significant amounts of urea and inorganic ions in urine. Previous studies have shown that H-NS plays a significant role in NaCl induced changes in osmolarity. However, our results demonstrate that urea, but not NaCl, mediate activation of P3 independently of the transcriptional regulator TyrR. Indeed, a previous gene array study also revealed that *usp* was upregulated in the presence of urea [63]. We therefore speculate on the possible existence of another, TyrR and H-NS independent mechanism, regulating expression from P3, both in *E. coli* and *S. bongori*. In UPEC strains, the presence of urea in urine could trigger upregulation of *usp* expression in the urinary tract.

In conclusion, our results highlight the complex regulation of the *usp* gene encoded by PAI*usp.* We further demonstrate that *usp* is a novel member of the TyrR and H-NS regulons due to its integration into the *aroP pdhR* intergenic region where the *aroP* P3 promoter drives its expression. TyrR and H-NS play antagonistic roles in regulating *usp* expression, with *usp* P3 promoter activity stimulated by TyrR and repressed by H-NS. In the host, various environmental factors such as aromatic amino acids, temperature as well as urea could induce significant increases in *usp* expression. Following acquisition of PAI*usp* by a common ancestor, species divergence and subsequent mutations in the *usp* P3 promoter region fine-tuned expression in the bacterial hosts.

## Figures and Tables

**Table 1:** The bacterial strains and plasmids used in this study.

Strains or Plasmids	Relevant Charasteristic(s) ^#^	Reference
Strains		
*E. coli* DH5α	Cloning host; F– Φ80*lac*ZΔM15 Δ(*lac*ZYA-argF) U169 *rec*A1 *end*A1 *hsd*R17 (rK−, mK+) *pho*A *sup*E44 λ– *thi*-1 *gyr*A96 *rel*A1	Life Technologies
*E. coli* BL21 (DE3)	F^−^*omp*T *hsd S*_B_(r_B_^−^m_B_^−^) *dcmgal*λ (DE3)	Invitrogen
*E. coli* JW1889	*F-Δ(araD-araB)567*, *ΔlacZ4787*(::rrnB-3), *λ^−^*, *ΔaraF751::kan*, *rph-1*, *Δ(rhaD-rhaB)568*, *hsdR514*	[27]
*E. coli* JW1316 *E. coli* JW1225	*F-Δ(araD-araB)567*, *ΔlacZ4787*(::rrnB-3), *λ^−^*, *ΔtyrR760::kan*, *rph-1*, *Δ(rhaD-rhaB)568*, *hsdR514* *F-Δ(araD-araB)567*, *ΔlacZ4787*(::rrnB-3), *λ^−^*, *Δhns746:: kan*, *rph-1*, *Δ(rhaD-rhaB)568*, *hsdR514*	[27]
*E. coli* TA211	wild-type strain (UPEC isolate)	IMI
*E. coli* MG1655	wild-type strain	This laboratory
*S.bongori* NCTC 12419	wild-type strain	ATTC
Plasmids		
pRW50	Tc^R^; Low-copy-number promoterless *lac*Z trancriptional fusion vector	[28]
pRWBP	Tc^R^; 403 bp *Eco*RI-*Hind*III fragment from *S. bongori* NCTC 12419 *aro*P-*usp* intergenic region: *usp*-*lac*Z transcription fusion	This study
pRWBPM1	Tc^R^; pRWBP derivate; P3 (−35) mutation: CACTCT-->CA**AG**CT	This study
pRWBPM3	Tc^R^; pRWBP derivate; P3 (−10) mutation: TGGTAT-->T**TAATG**	This study
pRCPL	Tc^R^; 527 bp *Eco*RI-*Hind*III fragment from *E. coli* TA211 *aroP-usp* intergenic region: *usp-lacZ* trancription fusion	This study
pRWC10	Tc^R^; pRCPL derivate; P3 (−10) mutation: TGGTAT-->T**TAATG**	This study
pRWC35	Tc^R^; pRCPL derivate; P3 (−10) mutation: TGGTAT-->T**TAATG**	This study
pRWMGA-	Tc^R^; 215 bp *Eco*RI-*Hind*III fragment from *E. coli* K-12 MG1655*aroP*-*pdhR* intergenic region: *aroP*-*lac*Z transcription fusion	
pJCAT	Ap^R^; High-copy-number promoterless *cat* trancription fusion vector (pJET1.2 cloning vector (Thermo Fisher Scientific) derivate)	This study
pJBLC	Ap^R^; 403 bp *Pst*I-*Bst*EII fragment from *S. bongori aroP-usp* intergenic region: *usp-cat* transcription fusion	This study
pJBSC	Ap^R^; pJBLC derivate; 247 bp fragment without both TyrR binging boxes	This study
pJBTC	Ap^R^; pJBLC derivate; 315 bp fragment without weak TyrR binding box	This study
pJBTD2	Ap^R^; 111 bp *Pst*I-*Bst*EII fragment from *S. bongori aroP-usp* intergenic region only with strong TyrR box and P3 promoter	This study
pJBTD3	Ap^R^; pJBTD2 derivate: 111 bp fragment extended for 64 bp	This study
pET8HT	Ap^R^; pET8c -derived expression vector with P_T7_ promoter: expression and production of recombinant N-terminally His_6_-tagged TyrR	This study
pET9HT	Ap^R^; pET8c -derived expression vector with P_T7_ promoter: expression and production of recombinant N-terminally His_6_-tagged H-NS	This study
pUSP4R-L	Ap^R^; T7 promoter of pUSP4 replaced with native *E. coli* TA211 *usp* promoter.	[14]
pCUPBI	Ap^R^; T7 promoter of pUSP4 replaced with native *S. bongori* NCTC 12419 promoter	This study

^#^—abbreviations: Tc, tetracycline; Ap, ampicillin; R, resistance; ATCC, American Type Culture Collection; IMI, Institute of microbiology and immunology, Medical Faculty, University of Ljubljana, Ljubljana, Slovenia.

**Table 2:** Primer sequences used in this study.

Primer	Sequence (5′→3′)	Purpose
Cm_Bst_F	TTGGTAACCAAGAGAAAAAAATCACTGGATATAC	PCR amplification of *cat* gene from pLysS plasmid for cloning in pJBPLN3 (intermediate plasmid)
Cm_Xba_R	TTTCTAGATTACGCCCCGCCCTGCCACTCAT	
Bong_L_Pst_F	CTGCAGCATAAAACCTCGTGTATGGTGCGC	PCR amplification of *S. bongori aroP-usp* promoter region for cloning in pJBLC
Bong_S_Pst_F	CTGCAGCACTATCACTCTTTTGTCTTGGTAAG	PCR amplification of *S. bongori aroP-usp* promoter region for cloning in pJBSC and also used in EMSA
Bong_L_Bst_R	GGTTACCCATTTTACGACATCCGAATATAAATTAAACGCC	PCR amplification of *S. bongori aroP-usp* promoter region for cloning in pJBLC, pJBSCm, pJBTCm
Bong_promS_tyr_F	TTCTGCAGCTTTTGTATTGATTTATTTACAGTGA	PCR amplification of *S. bongori aroP-usp* promoter region for cloning in pJBTC, pJBTD2 and pJBTD3
Bong_prom_del2_R	TTGGTTACCCATTTTACGACATCCGAACTCGTCATCACCCCTGCACAAAGCAGGTGCAT	PCR amplification of *S. bongori aroP-usp* promoter region for cloning in pJBTD2
Bong_prom_del3_R	TTGGTTACCCATTTTACGACATCCGAATGCAAAATAAAGTGAGTAATGCTAAAAGGG	PCR amplification of *S. bongori aroP-usp* promoter region for cloning in pJBTD3
Bong_L_Eco_F	GAATTCATAAAACCTCGTGTATGGTGCGC	PCR amplification of *S. bongori aroP-usp* promoter region for cloning in pRWBP, pRWBPM1, pRWBPM2 and also used in EMSA
Mut10_Vsp	TGTCTTGGTAAGAATAATTAATTGTGACTGCGAATGCAC	PCR amplification of *S. bongori aroP-usp* promoter region for cloning in pRWBP, pRWBPM1, pRWBPM2
Mut35_Hind	ATTAAATATCACTATCAAGCTTTTGTCTTGGTAAGAA	PCR amplification *of S. bongori aroP-usp* promoter region for cloning in pRWBP, pRWBPM1, pRWBPM2
Bong_prom_Hind_R	TTAAGCTTTACGACATCCGAATATAAATTAAACGCC	PCR amplification of *S. bongori aroP-usp* promoter region for cloning in pRWBP, pRWBPM1, pRWBPM2 and also used in EMSA
Coli_L_Eco_F	TTGAATTCTATGAAACCTCGTGTTGTGTTGTT	PCR amplification of *E. coli* 211 *aroP-usp* promoter region for cloning in pRCPL, pRWC10 and pRWC35
Mut_coli_10_Vsp	ACTATCACAAGATAAATTAATATGCGCGAGAATGCACC	
Mut_coli_35_Hind	ACTGAATTATGATGATAAAGCTTTCAACTATCACAAG	
Coli_L _R_Hind	TTAAGCTTTATTTCATTCCCGTTTATCCTTAAC	
MG_prom_F_Eco	TTGAATTCGAAACCTCGTGCGGTGGTTGTTTTTTTG	PCR amplification of *E. coli* K-12 MG1655 *aroP* promoter region for cloning in pRWMGA-
MG_prom_R_Hind	TTAAGCTTTACAAACGGTTTCTTTTTAAGC	
TyrR_Bam_F	TTGGATCCCGTCTGGAAGTCTTTTGTGAAGACC	PCR amplification of *tyrR* gene from *E. coli* DH5α for cloning in pET8HT PCR amplification of *hns* gene from *E. coli* DH5α for cloning in pET9HT
TyrR_Mlu_R H-NS Bam F H-NS Mlu R	TTACGCGTTACTCTTCGTTCTTCTTCTGACTCAGAC TTGGATCCAGCGAAGCACTTAAAATTCTGAAC TTACGCGTTTATTGCTTGATCAGGAAATCGTCG	

## Supplementary Materials

The following are available online at https://www.mdpi.com/2076-2607/8/3/330/s1, Figure S1: Usp homologs encoded by PAI*usp*, Figure S2: Isolation of *E. coli* strain conveying high P3 promoter activity on MacConkey agar plates, Table S1: PAI*usp* containing strains identified by PSI-BLAST and tBLASTn analysis, excluding *E. coli* strains; Table 2: Usp levels from *E. coli* and *S. bongori usp* promoters in w.t. *E. coli* and isogenic hns defective strain.
