# Supplementary material for: The Uropathogenic Specific Protein Gene usp from Escherichia coli and Salmonella bongori is a Novel Member of the TyrR and H-NS Regulons
Source: Microorganisms. 2020 Feb 26;8(3):330. doi: 10.3390/microorganisms8030330 (PMC7142922; doi:10.3390/microorganisms8030330)
Supplement: Supplementary file 1 [file microorganisms-08-00330-s001.pdf]

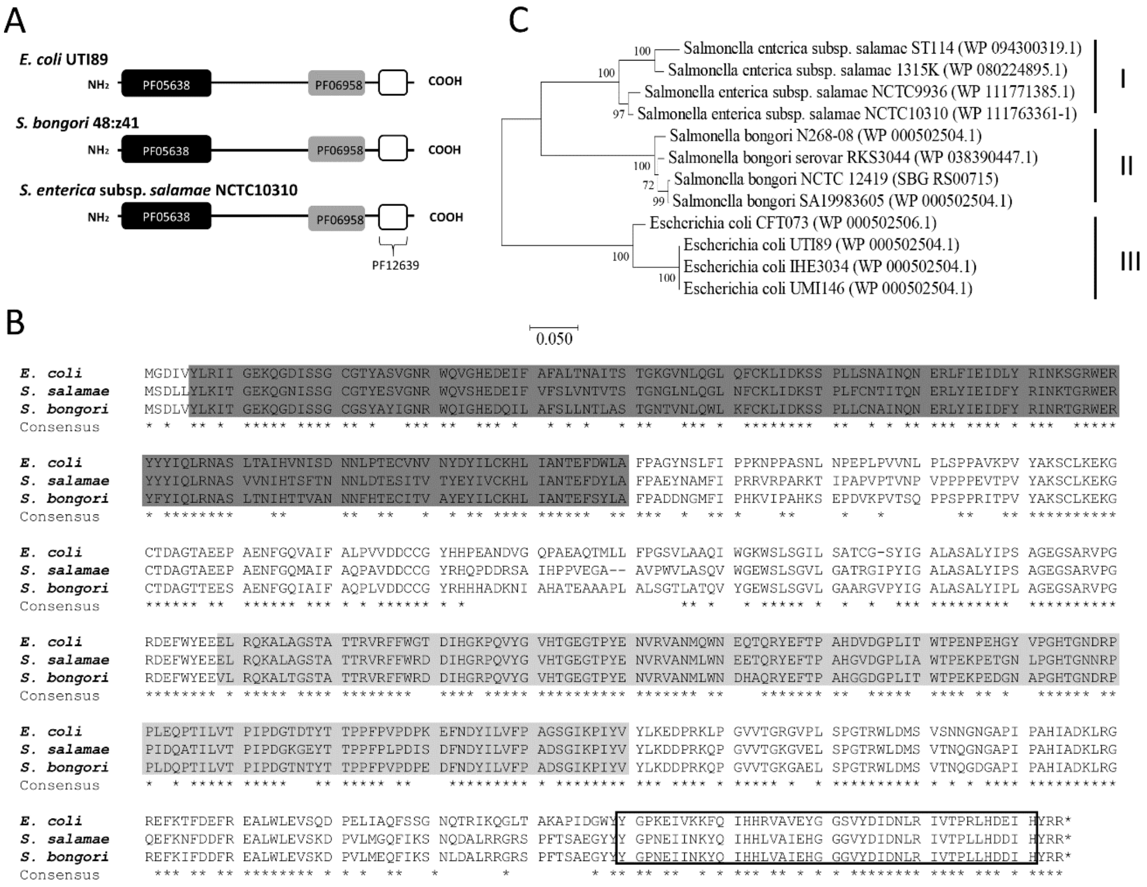

Figure S1. Usp homologs encoded by PAI*usp*. (A) Schematic representation of Usp proteins encoded by PAI*usp* from *E. coli* UTI89 (Accession no. WP_000502504.1), *S. bongori* serovar 48:z4:-- str. RKS3044 (Accession no. WP_038390447.1) and *S. enterica* subsp. *salamae* NCTC10310 (Accession no. WP_111763361.1). Black, light grey and white boxes represent conserved domains PF05638 (Hcp domain), PFO6958 (S-type pyocin domain) and PF12639 (colicin nuclease domain), respectivly. (B) Protein sequence comparison between Usp homologs of *E. coli* UTI89, *S. bongori* serovar 48:z4:-- str. RKS3044 and *S. enterica* subsp. *salamae* NCTC10310. Usp protein sequences were aligned using the multiple sequence alignment tool ClustalW. Identical and conserved residues are indicated below the alignment by an asterix. Dark grey, light grey and white boxes represent Hcp, S-type and colicin nuclease domain, respectively. (C) The neighbor-joining phylogenetic tree (bootstrap n = 1000; Poisson correction; Complete gap deletion; Midpoint rooted) showing the phylogenetic relationship between the Usp proteins from various species. Clades are indicated by large Roman numerals.

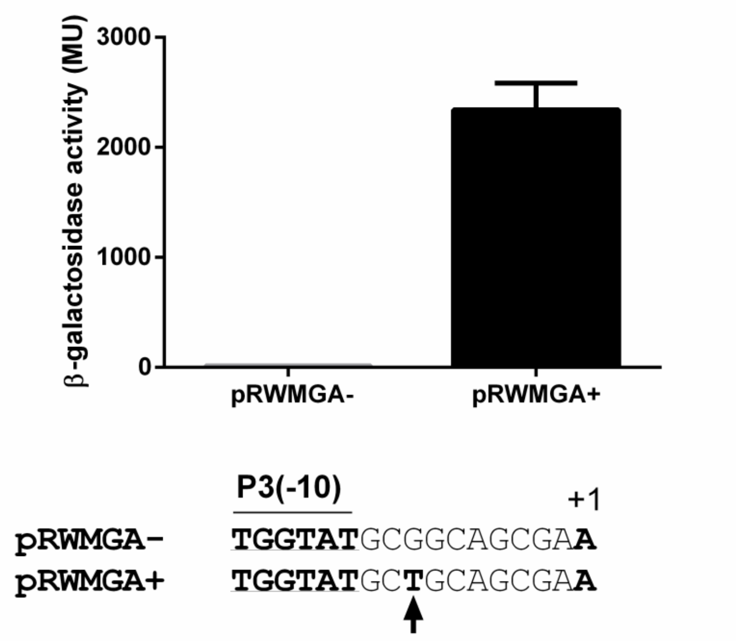

Figure S2. Isolation of *E. coli* strain conveying high P3 promoter activity on MacConkey agar plates. *E. coli* containing pRWAro- plasmid (*aroP* P3 promoter from *E. coli* K-12 MG1655 which does not harbor PAI*usp*, fused with *lac*Z gene) was plated on MacConkey agar. Remarkably, within 2-3 days of growth we observed some red colonies from which plasmid DNA was isolated and the promoter region sequenced promoter. Surprisingly, sequencing revealed a single modification in the discriminator region; -8G to T substitution, relative to the transcription start point (the new plasmid was termed pRWAro+). Subsequently, we compared non-mutated, wild type (pRMGA-) and modified (pRMGA+) promoter P3 activity on the basis of β-galactosidase activity. Units for β-galactosidase assay are those defined by Miller, 1974. Error bars represent standard deviations of the means of two independent experiments. Position of the spontaneous mutation is indicated with an arrow.

Table S1 PAI*usp* containing strains identified by PSI-BLAST and tBLASTn analysis, excluding *E. coli* strains.

Organisms containing PAIusp	Strain	Genome RefSeq ID	Usp protein accession number or locus tag
Salmonella bongori	NCTC 12419	NC_015761.1	SBG_RS00715
Salmonella bongori	N268-08	NC_021870.1	AGR57335.1
Salmonella bongori serovar 48:z41:--	RKS3044	NZ_CP006692.1	WP_038390447.1
Salmonella bongori serovar 66:z41:-	SA19983605	NZ_CP022120.1	LFZ56_RS17695
Salmonella bongori serovar 44:z39:-	BCW_1554	NZ_MXOF01000001.1	WP_079777515.1
Salmonella enterica subsp. *salamae* serovar 57:z29:z42	ST114	NZ_CP022467.1	WP_094300319.1
Salmonella enterica subsp. *salamae*	NCTC9930	NZ_LS483456.1	DQN98_RS21100
Salmonella enterica subsp. *salamae*	RKS2993	NZ_JXTT01000055	WP_046094099.1
Salmonella enterica subsp. *salamae* serovar Greenside	NCTC9936	NZ_LS483475.1	WP_111771385.1
Salmonella enterica subsp. *salamae*	NCTC10310	NZ_LS483477.1	WP_111763361.1
Salmonella enterica subsp. *salamae* serovar 55:k:z39	1315K	NZ_CP022139.1	WP_080224895.1
Salmonella enterica subsp. *salamae* serovar 58:d:z6	327-85	NZ_MXLI01000001.1	WP_079805736.1
Salmonella enterica subsp. *salamae* serovar 50:b:z6	BCW_1520	NZ_MXPI01000002.1	WP_079818387.1
Salmonella enterica subsp. *salamae* serovar 48:d:z6	BCW_1519	NZ_MXPJ01000002.1	WP_079814527.1
Salmonella enterica subsp. *salamae*	NCTC10436	NZ_LS483428.1	WP_111753446.1
Salmonella enterica subsp. *salamae*	NCTC9948	LS483495.1	SQJ44863.1
Salmonella enterica subsp. *salamae*	NCTC9930	LS483456.1	SQH95318.1

Strains or plasmids	Relevant charasteristic(s)[a]	Reference
Strains		
E. coli DH5α	Cloning host; F– Φ80*lac*ZΔM15 Δ(*lac*ZYA-argF) U169 *rec*A1 *end*A1 *hsd*R17 (rK–, mK+) *pho*A *sup*E44 λ– *thi*-1 *gyr*A96 *rel*A1	Sambrook *et al.* (1989)
E. coli BL21 (DE3)	F^- *omp*T *hsd* $S_B(r_B^- m_B^-)$ *dcm galλ* (DE3)	Promega
E. coli JW1889	*F-Δ(araD-araB)567*, *ΔlacZ4787*(::rrnB-3), *λ*$^-$, *ΔaraF751::kan*, *rph-1*, *Δ(rhaD-rhaB)568*, *hsdR514*	Baba *et al.* (2006)
E. coli JW1316	*F-Δ(araD-araB)567*, *ΔlacZ4787*(::rrnB-3), *λ*$^-$, *ΔtyrR760::kan*, *rph-1*, *Δ(rhaD-rhaB)568*, *hsdR514*	Baba *et al.* (2006)
E. coli TA211	wild-type strain (UPEC isolate)	IMI
S.bongori NCTC 12419	wild-type strain	ATTC
Plasmids		
pRW50	Tc^R; Low-copy-number promoterless *lac*Z trancriptional fusion vector	Lodge *et al.* (1992)
pRWBP	Tc^R; 403 bp *Eco*RI-*Hind*III fragment from *S. bongori* NCTC 12419 *aro*P-*usp* intergenic region: *usp-lac*Z transcription fusion	This study
pRWBPM10	Tc^R; pRWBP derivate; P3 (-35) mutation: CACTCT-->CA**AG**CT	This study
pRWBPM35	Tc^R; pRWBP derivate; P3 (-10) mutation: TGGTAT-->T**TAATG**	This study
pRCPL	Tc^R; 527 bp EcoRI-HindIII fragment from *E. coli* TA211 *aroP-usp* intergenic region: *usp-lacZ* trancription fusion	This study
pRWC10	Tc^R; pRCPL derivate; P3 (-10) mutation: TGGTAT-->T**TAATG**	This study
pRWC35	Tc^R; pRCPL derivate; P3 (-10) mutation: TGGTAT-->T**TAATG**	This study
pJCAT	Ap^R; High-copy-number promoterless *cat* trancription fusion vector (pJET1.2 cloning vector (Thermo Fisher Scientific) derivate)	This study
pJBLC	Ap^R; 403 bp *Pst*I-*Bst*EII fragment from *S. bongori aroP-usp* intergenic region: *usp-cat* transcription fusion	This study
pJBSC	Ap^R; pJBLC derivate; 247 bp fragment withouth both TyrR binging boxes	This study
pJBTC	Ap^R; pJBLC derivate; 315 bp fragment withouth weak TyrR binding box	This study
pJBTD2	Ap^R; 111 bp PstI-BstEII fragment from S. bongori aroP-usp intergenic region only with strong TyrR box and P3 promoter	This study
pJBTD3	Ap^R; pJBTD2 derivate: 111 bp fragment extended for 64 bp	This study
pJCLCH	Ap^R; High-copy number plasmid with N-terminally His_6-tagged Cat under *E.coli* TA211 *usp* promoter	This study
pET8TH	Ap^R; pET8c -derived expression vector with P_{lacUV5} promoter: expression and production of recombinant N-terminally His_6-tagged TyrR	This study

[a] - abbreviations: Tc, tetracycline; Ap, ampicillin; R, resistance; ATCC, American Type Culture Collection; IMI

Table 2. Usp levels from *E. coli* and *S. bongori usp* promoters in w.t. *E. coli* and isogenic *hns* defective strain.

E. coli		
25°C	w.t.	2.0
	hns^{-}	35.4
37°C	w.t	9.4
	hns^{-}	26.4
42°C	w.t.	9.4
	hns^{-}	34

S. bongori		
25°C	w.t.	0.24
	hns^{-}	1.5
37°C	w.t	2.0
	hns^{-}	14
42°C	w.t.	1.9
	hns^{-}	24

Top, Usp from *E. coli* promoter; 10^8 cells (w.t. and *hns*- defective) grown at various temperatures. Bottom, Usp from *S. bongori* promoter; 10^8 cells (w.t. and *hns*- defective) grown at various temperatures. .
